# Nephrocalcinosis tendency does not worsen under burosumab treatment for X-linked hypophosphatemic rickets: a multicenter pediatric study

**DOI:** 10.3389/fped.2024.1487890

**Published:** 2024-12-02

**Authors:** Shelly Levi, Daniel Landau, Miriam Davidovits, Mika Shapira Rootman, Avivit Brener, Shoshana Gal, Yael Borovitz, Ori Goldberg, Rachel Bello, Roxana Cleper, Yael Lebenthal, Yael Levy-Shraga, Dov Tiosano, Adi Chezana, Ravit Regev, Leonid Zeitlin

**Affiliations:** ^1^ Pediatric Nephrology Institute, Schneider Children’s Medical Center, Petach Tikva, Israel; ^2^ The Faculty of Medicine, Tel-Aviv University, Tel-Aviv, Israel; ^3^ Department of Medical Imaging, Rambam Medical Health Care Campus, Haifa, Israel; ^4^ Pediatric Endocrinology and Diabetes Unit, Dana-Dwek Children’s Hospital, Tel Aviv Sourasky Medical Center, Tel Aviv, Israel; ^5^ Division of Pediatric Endocrinology, Ruth Rappaport Children’s Hospital, Rambam Medical Health Care Campus, Haifa, Israel; ^6^ Bruce Rappaport Faculty of Medicine, Technion, Haifa, Israel; ^7^ Institute of Pulmonology, Schneider Children’s Medical Center, Petach Tikva, Israel; ^8^ Pediatric Pulmonary Service, Faculty of Medicine, Hebrew University, Jerusalem, Israel; ^9^ The Jesse Z and Sara Lea Shafer Institute for Endocrinology and Diabetes, National Center for Childhood Diabetes, Schneider Children’s Medical Center, Petach Tikva, Israel; ^10^ Pediatric Nephrology Unit, Dana-Dwek Children’s Hospital, Tel Aviv Sourasky Medical Center, Tel Aviv, Israel; ^11^ Pediatric Endocrinology Unit, The Edmond and Lily Safra Children’s Hospital, Chaim Sheba Medical Center, Tel-Hashomer, Israel; ^12^ Goldman School of Medicine, Faculty of Health Sciences, Ben Gurion University of the Negev, Be'er Sheva, Israel; ^13^ Pediatric Orthopedic Department, Dana-Dwek Children’s Hospital, Tel Aviv Sourasky Medical Center, Tel-Aviv, Israel

**Keywords:** XLH, FGF23, burosumab, hypercalciuria, nephrocalcinosis

## Abstract

**Background:**

X-linked hypophosphatemic rickets (XLH) is associated with uninhibited FGF23 activity, which leads to phosphaturia, hypophosphatemia and depressed active vitamin D (1,25OH2D) levels. Conventional treatment with phosphate supplements and vitamin D analogs may lead to hypercalciuria (HC), nephrocalcinosis (NC) and hyperparathyroidism. We investigated the effects of burosumab treatment, an anti-FGF23 monoclonal antibody recently approved for XLH, on these complications.

**Methods:**

This retrospective study included children with XLH who were treated with burosumab for at least one year at one of three referral centers. Clinical and biochemical potential treatment outcomes were regularly followed, including multiple urine calcium measurements and NC severity score (0 = no NC, 3 = worse NC).

**Results:**

Twenty-six (13 male) children aged 7.6 ± 3.9 years were followed for 27.5 ± 9.6 months. Mean serum phosphate levels rapidly increased from 2.67 ± 0.61 at baseline to 3.57 ± 0.53 mg/dL after 3 months (*p* < 0.001) and remained stable thereafter. Concomitant decreases were observed in phosphaturia, serum alkaline phosphatase and parathyroid hormone. HC (U-Ca/Cr > 0.2 mg/mg) was detected in 2/26 (7.7%) patients before burosumab initiation, resolved in one and persisted, albeit improved, in the second. Two patients were newly diagnosed with HC, 15 and 3 months after therapy, which persisted in one of them despite dose reduction attempts. Seven patients had NC at baseline (mean score: 1.8 ± 0.34), but none showed deterioration or developed new NC.

**Conclusion:**

In children with XLH treated with burosumab, HC was an infrequent side effect and preexisting NC did not worsen.

## Introduction

X-linked hypophosphatemic rickets (XLH) is the most common heritable form of rickets, with an incidence of approximately 3.9 per 100,000 live births (1). XLH results from a loss-of-function mutation in the PHEX gene (phosphate-regulating endopeptidase homolog X-linked), leading to overproduction of fibroblast growth factor 23 (FGF23) by osteoblasts (2, 3). Elevated FGF23 levels cause phosphaturia and hypophosphatemia (4) by the direct suppression of NaPi-2a and NaPi-2c cotransporters in proximal tubular cells, or by affecting parathyroid hormone (PTH) activity (5). In addition, by inhibiting the activity of kidney 1-alpha-hydroxylase and stimulating that of 24- hydroxylase, FGF23 diminishes the production of active vitamin D metabolite [calcitriol, 1,25(OH)2D] and increases its catabolism, respectively, contributing to impaired intestinal phosphate and calcium absorption (6).

Clinically, XLH manifests in early childhood with rickets, growth retardation, limb deformities, pain, and physical disability (3).

Traditional treatment involves daily oral phosphate and active vitamin D analogs, but this treatment is associated with incomplete resolution of rickets, residual skeletal deformities, persistent short stature, and gastrointestinal side effects (7). Significant long-term complications of this therapy include: hyperparathyroidism (secondary and even tertiary) in 62%–87%; hypercalciuria (HC) and nephrocalcinosis (NC) in up to 60% of pediatric patients (7, 8).

Burosumab, a recombinant human IgG1 monoclonal antibody targeting FGF23 has been introduced as a treatment option since 2018 (9). This treatment improves phosphate levels and bone healing secondary to restoration of tubular phosphate reabsorption and increased serum 1,25(OH)2D levels (10–12). However, the possible increase in 1,25(OH)2D due to FGF23 suppression raises concerns about elevation in serum and urine calcium levels, leading to (or worsening) renal calcium deposition (NC). Current guidelines recommend regular monitoring of biochemical profiles including urine calcium secretion and renal ultrasound to detect NC (13). However, data on risk of increased urinary calcium excretion and kidney calcium deposition are still lacking. The aim of this study was to evaluate the extent of adverse kidney effects, including HC and NC/nephrolithiasis, under burosumab treatment.

## Patients and methods

### Study design and treatment

We conducted a multicenter retrospective longitudinal study of pediatric patients with XLH who had been treated with burosumab for at least 1 year by the time of inclusion. The study was carried out at three tertiary centers in Israel: Schneider Children's Medical Center of Israel, Dana-Dwek Children's Hospital at the Tel Aviv Sourasky Medical Center, and the Ruth Rappaport Children's Hospital, Rambam Health Care Campus. Inclusion criteria were age under 18 years; a diagnosis of XLH based on clinical, biochemical, radiological criteria (2), and a confirmed pathogenic variant in PHEX gene. Only patients with available kidney sonography imaging at baseline and at least one-year post-treatment were included. Some of the patients included in this study have been previously described as part of a larger cohort of XLH patient, where the effects of burosumab (with or without growth hormone) on growth have been described (14). The baseline point for the study period was determined as the start of burosumab treatment.

Patients diagnosed before 2019, when burosumab was not widely available in our country, were treated with conventional treatment with phosphate supplements and active vitamin D (one alpha D3) as well as vitamin D supplementation to achieve normal (sufficient) serum levels of above 30 ng/mL (15). Phosphate supplements and active vitamin D were withdrawn two weeks before the initiation of burosumab. Nutritional consultation was provided, advising calcium intake according to age-specific recommendations. Burosumab was then administered according to the recommended protocol, starting at 0.4–0.8 mg/kg every 2 weeks, with dose adjustments to maintain serum phosphate within the low to mid-normal range for age (2).

### Study assessments

Baseline assessments included physical examination, anthropometric measurements, and laboratory evaluations, which were repeated 2 weeks after treatment initiation or dose adjustments, every 3 months during the first year of treatment, and at least every 6 months thereafter. Laboratory tests included measured serum calcium, phosphate, creatinine, alkaline phosphatase (ALKP), PTH, 25(OH)-, and 1,25(OH)2D. Spot urine samples, as advised at published guidelines (2), were analyzed for calcium, phosphate, and creatinine, collected under fasting conditions in most patients. X-rays for rickets evaluation were performed at baseline and then every 6–12 months as needed. All patients were referred to kidney ultrasound before burosumab initiation, every 6 months during the first year of burosumab treatment, and yearly thereafter.

Anthropometric data were converted to sex and age-specific *z*-scores using the CDC2000 Growth Charts (16). HC was defined as at least two urinary spot tests of calcium to creatinine (Ca/Cr) ratio above age-specific norms (17, 18). Patients with HC were advised on proper hydration and low sodium diet. Tubular phosphate reabsorption (TPR) and tubular maximum reabsorption of phosphate for glomerular filtration rate (TmP/GFR) (10) were calculated. Serum creatinine was measured using either the modified Jaffe or enzymatic method, depending on the medical center. Glomerular filtration rate (GFR, mL/min/1.73 m^2^) was estimated by the Schwartz formula, using a coefficient of 0.45–0.55 for the Jaffe method or 0.413 for the enzymatic creatinine assessment respectively (19, 20). Rickets severity was assessed using the Thacher Rickets Severity Score (RSS) ranging from 0 (no rickets) to 6 (severe rickets), for wrists and knees separately. We used lower extremity radiographs only, as wrist radiograph were not part of the routine care (21). Kidney ultrasounds for NC were retrospectively graded by a single experienced radiologist (M.S.R.), who was blinded to the patients’ information, using a published score (22), where 0 indicates no NC and 3 indicates homogeneous increase in the echogenicity of the entire medullary pyramid.

The study was approved by the Institutional Research Ethics Board of all the participating centers.

### Statistical analysis

In this study, cumulative data are presented as mean and standard deviation (SD) or mean and 95% confidence intervals (CI), median and interquartile range or as frequency and percentage. The statistical significance of the repeated continuous pharmacodynamic markers over time was evaluated using a paired Student's *t*-test, comparing the markers to their pretreatment or baseline state. Categorical variables were compared using chi-square test. A two-tailed *p*-value <0.05 was considered statistically significant. IBM SPSS statistical package (version 24, Chicago, IL, USA) was used for all statistical analyses. Pearson correlation coefficient between baseline urinary Ca/Cr ratio and values obtained after 12 and 24 months were calculated for all available pairs.

## Results

### Burosumab doses and duration and it's effect on growth

A total of 30 children had been treated with burosumab for at least one year at the time of data analysis, 4 children were excluded due to lack of kidney ultrasound data, therefore 26 met the inclusion criteria. Baseline patients’ characteristics are summarized on Table 1. Most patients were treated with conventional treatment prior to burosumab initiation. The mean (±SD) follows up duration under burosumab treatment was 28.4 ± 9.4 months. The mean burosumab dose (administered every 2 weeks) at 6, 12, 24 and 36 months was 1.1 ± 0.44, 1.07 ± 0.4, 0.94 ± 0.39 and 1.03 ± 0.44 mg/kg, respectively.

**Table 1:** Baseline patient characteristics.

Number of patients	26
Male, *n* (%)	13 (50)
Age at inclusion, years (mean ± SD)	7.7 ± 3.9
Positive family history of XLH, *n* (%)	17 (65)
Previously treated with conventional therapy, *n* (%)	24 (92)
Conventional therapy duration, years (mean ± SD)	5.4 ± 3.9
Mean (±SD) burosumab dose, mg/kg	1.03 ± 0.42
Maximal burosumab dose, mg/kg (mean ± SD)	1.2 ± 0.5
Burosumab treatment length, months (mean ± SD)	28.4 ± 9.4

The mean height *z* scores were −1.6 ± 1.25 and −1.6 ± 1 at 12 months pretreatment and at baseline, and −1.46 ± 1, −1.6 ± 0.85, and −1.4 ± 1 at 12, 24 at 30 months follow up respectively (*p* = NS). RSS decreased from 1.76 ± 0.97 at baseline (*n* = 13) to 0.57 ± 0.58 (*n* = 14) and 0.37 ± 0.6 (*n* = 12) at 12- and 24-months post treatment, respectively (*p* < 0.001).

### Effect of burosumab treatment on metabolic parameters

**Figure 1:**
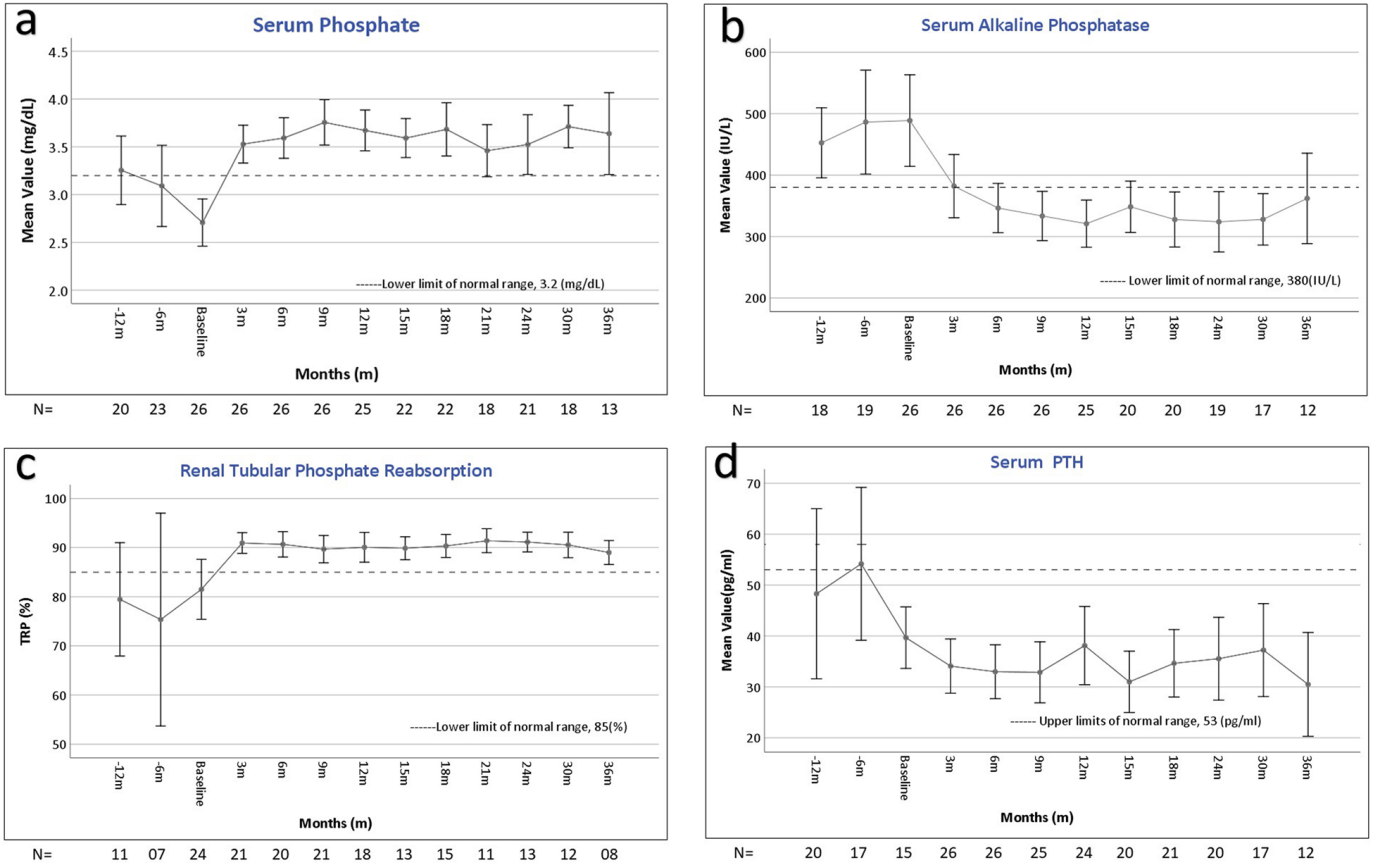
Changes in laboratory parameters during the course of burosumab therapy. Error bars represent the mean and 95% confidence interval values at different times points of serum phosphate **(a)**, serum alkaline phosphatase **(b)**, total reabsorption of phosphate (%) **(c)**, and parathyroid hormone (PTH) **(d)**, before, at baseline, and during the 36 months after the initiation of burosumab therapy. The horizontal broken line shows the lower **(a,b)** and upper **(c,d)** limits of normal for each laboratory value. All the values improved, with statistical significance, compared to baseline **(a–c)**, and compared to pretreatment **(d)**. The number of patients (*n*) assessed at each time point appears below the *X* axis in each graph.

As shown in Figure 1, under burosumab treatment mean serum phosphate levels rapidly increased and ALKP decreased to normal levels, parallel to a decrease in urinary phosphate loss (expressed as increase in TRP). Along with TRP, TmP/GFR also increased from 2.2 ± 0.6 at baseline to 3.3 ± 0.6, 3.3 ± 0.6, 3.1 ± 0.5 and 3.2 ± 0.6 mg/dL at 6, 12, 24 and 36 months, respectively (*p* < 0.05). Total serum calcium level remained stable and within normal limits during the study period (data not shown). Mean serum PTH were at the high normal levels at 12- and 6 months pre-burosumab treatment, then decreased at baseline (two weeks after discontinuation of conservative therapy) and remained mid normal thereafter (Figure 1d). The amount of high abnormal PTH measurements on conventional therapy decreased significantly from 19.2% (12/62) to 8.1% (17/108) during burosumab treatment (*p* = 0.013).

Active vitamin D [1,25(OH)2D] levels, slightly increased, without statistically significance, under burosumab treatment: from 48.4 ± 14.2 pg/mL at baseline (*n* = 14), then 51.4 ± 14.7 (*n* = 19), 56.7 ± 20.1 (*n* = 12) and 55.6 ± 9.5 (*n* = 6) pg/mL at 12, 24 and 36 months, respectively.

**Figure 2:**
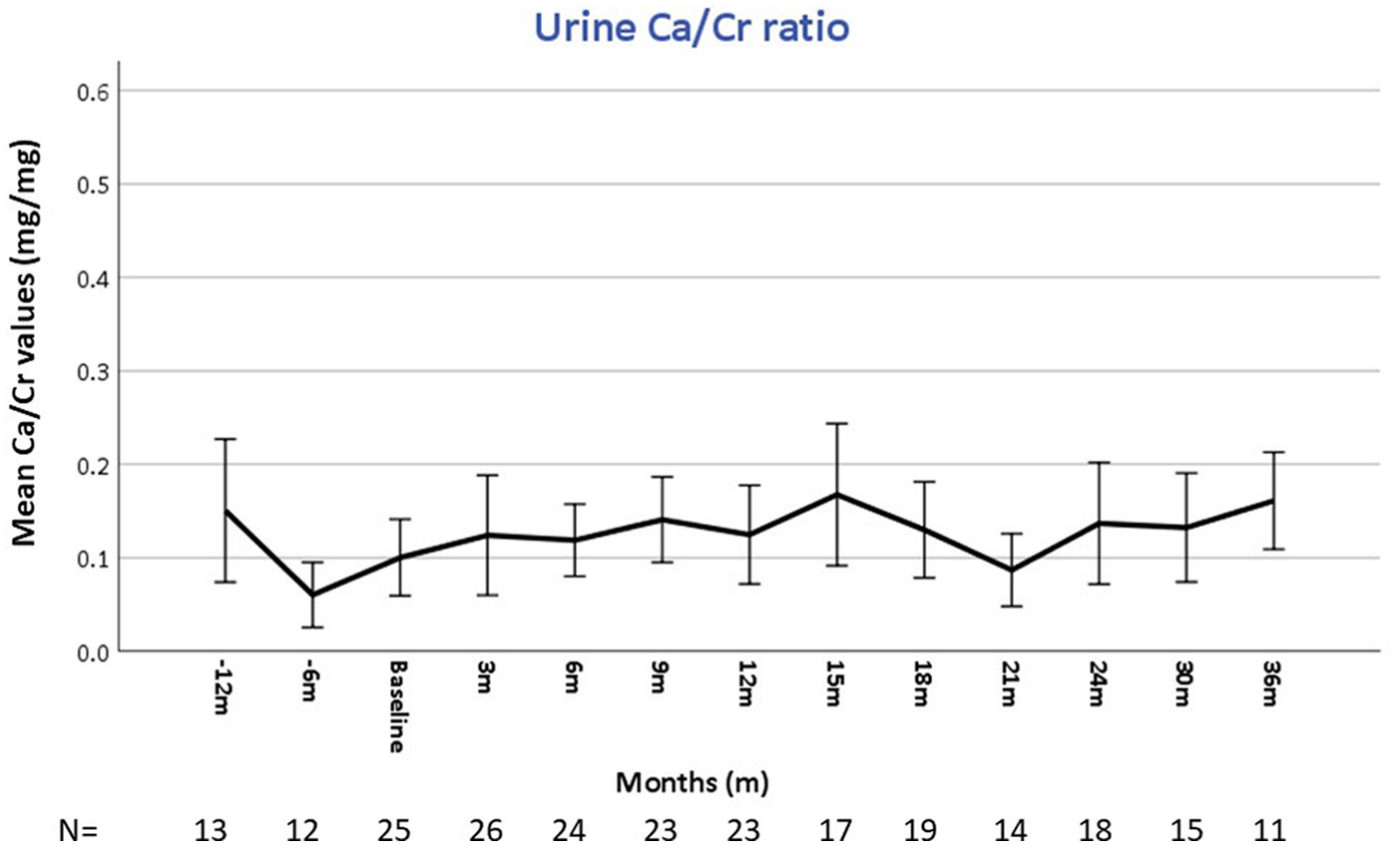
Mean urinary calcium to creatinine ratio during follow-up.

**Figure 3:**
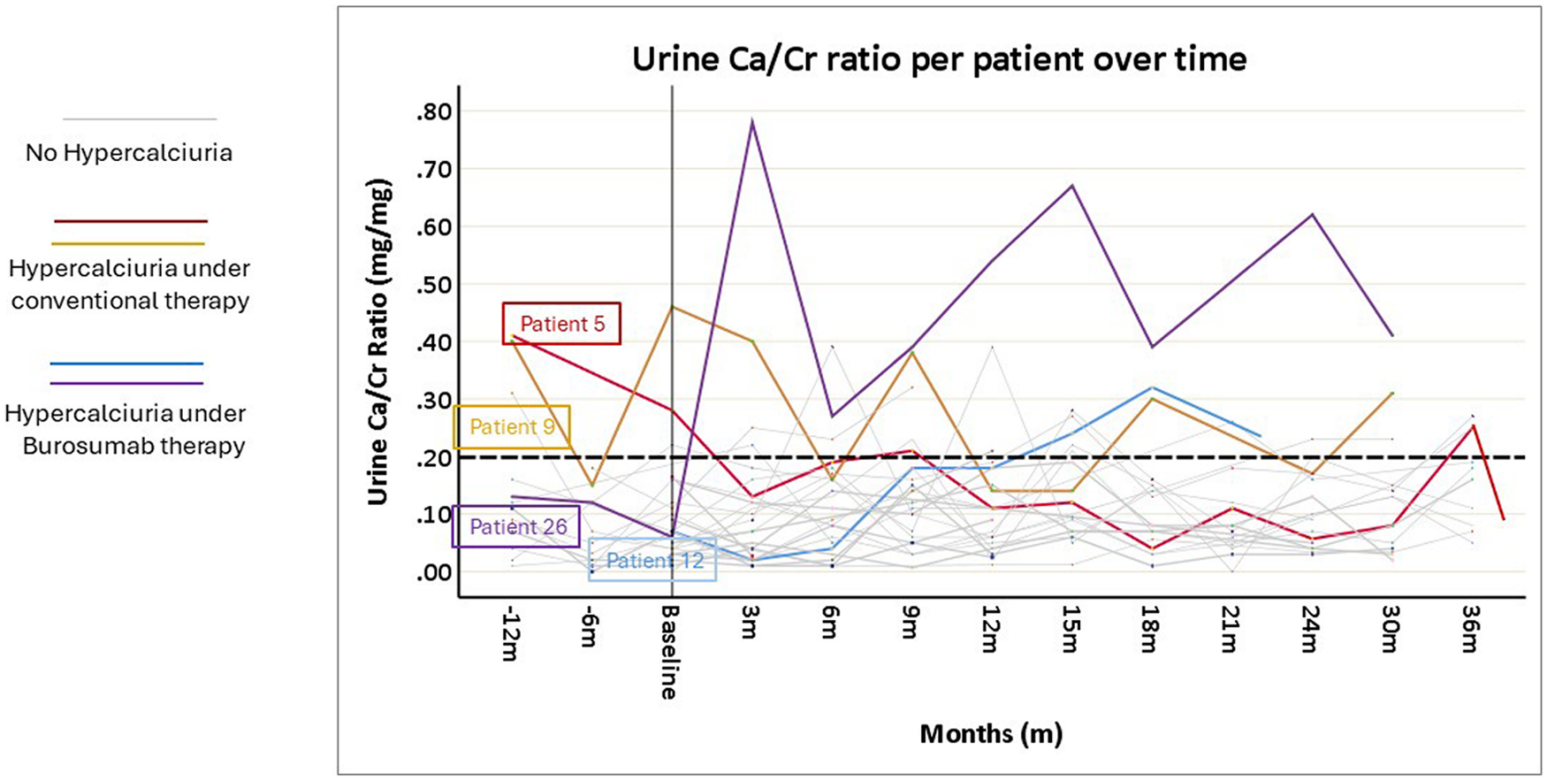
Spot urine calcium/creatinine (Ca/Cr) ratio values of each of the 26 included patients, before and after the initiation of burosumab treatment. The broken horizontal line shows the upper limit of normal Ca/Cr ratio (0.2 mg/mg). For details see the results section.

All patients underwent urine evaluations for Ca/Cr ratios at baseline and during the entire follow-up. The mean number of urinary samples per patient was 7.5 ± 2.4. Overall, mean urinary calcium was stable and within normal limits over the study period (Figure 2). Correlation coefficients calculated comparing baseline urine Ca/Cr ratio to values at 12- and 24-months post burosumab showed no significant trend or change from baseline values (*N* = 22, *r* = −0.017, *P* = 0.939, and *N* = 17, *r* = 0.046, *p* = 0.86, respectively). Urinary Ca/Cr ratios for each patient over the study period are presented on Figure 3. HC was present at baseline in 2/26 (patients 5 and 9, Figure 3). Their mean Ca/Cr ratios were 0.34 and 0.43 mg/mg within the year before burosumab treatment, at ages 8 and 6.5 years, respectively. Both received conventional treatment for 7.3 and 4.5 years. For patient 5, HC resolved after cessation of conventional treatment and did not recur during 36 months of follow up. For patient 9, mild HC persisted intermittently but gradually improved. In this patient, 24 h urine collection for calcium and citrate was also performed; with calcium excretion of 4 mg/kg/day 1- and 2- years post burosumab treatment and high citrate level of 1,077 mg/1.73 m^2^/day. New-onset HC was detected during burosumab therapy in two patients (number 12 and 26, Figure 3), both were females, after 15 months and 3 months from burosumab initiation, respectively. Patient 12 was treated with a burosumab dose of 1.7–1.9 mg/kg since age 2.5 years, without previous conventional therapy. She developed borderline HC at age 3.9 years: urine calcium/creatinine ratios were 0.24, 0.32, and 0.22 mg/mg at 15, 18, and 21 months after treatment onset, respectively. Urine collection, as well as urinary citrate testing, didn't performed in this patient. Her PTH and 1,25(OH)2D were normal. The second patient (number 26), started burosumab treatment at the age of 2.5 years, after only 6 months of conventional therapy. She developed HC after 3 months of treatment, at age 3 years, which persisted for a follow-up period of 30 months (mean U-Ca/Cr: 0.5 mg/mg, range: 0.27–0.78; *n* = 8). Her 24 h urine collection also revealed high urinary calcium secretion of 9.3 mgr/kg/day; with normal urinary citrate level (540 mg/1.73 m^2^/day). Following these results, burosumab dose was lowered gradually, from 1.3 to 0.53 mg/kg/dose, but HC persisted. Her PTH levels were normal in 9/10 measurements, and her 1,25(OH)2D levels were within normal limits.

### Renal imaging and outcome

**Figure 4:**
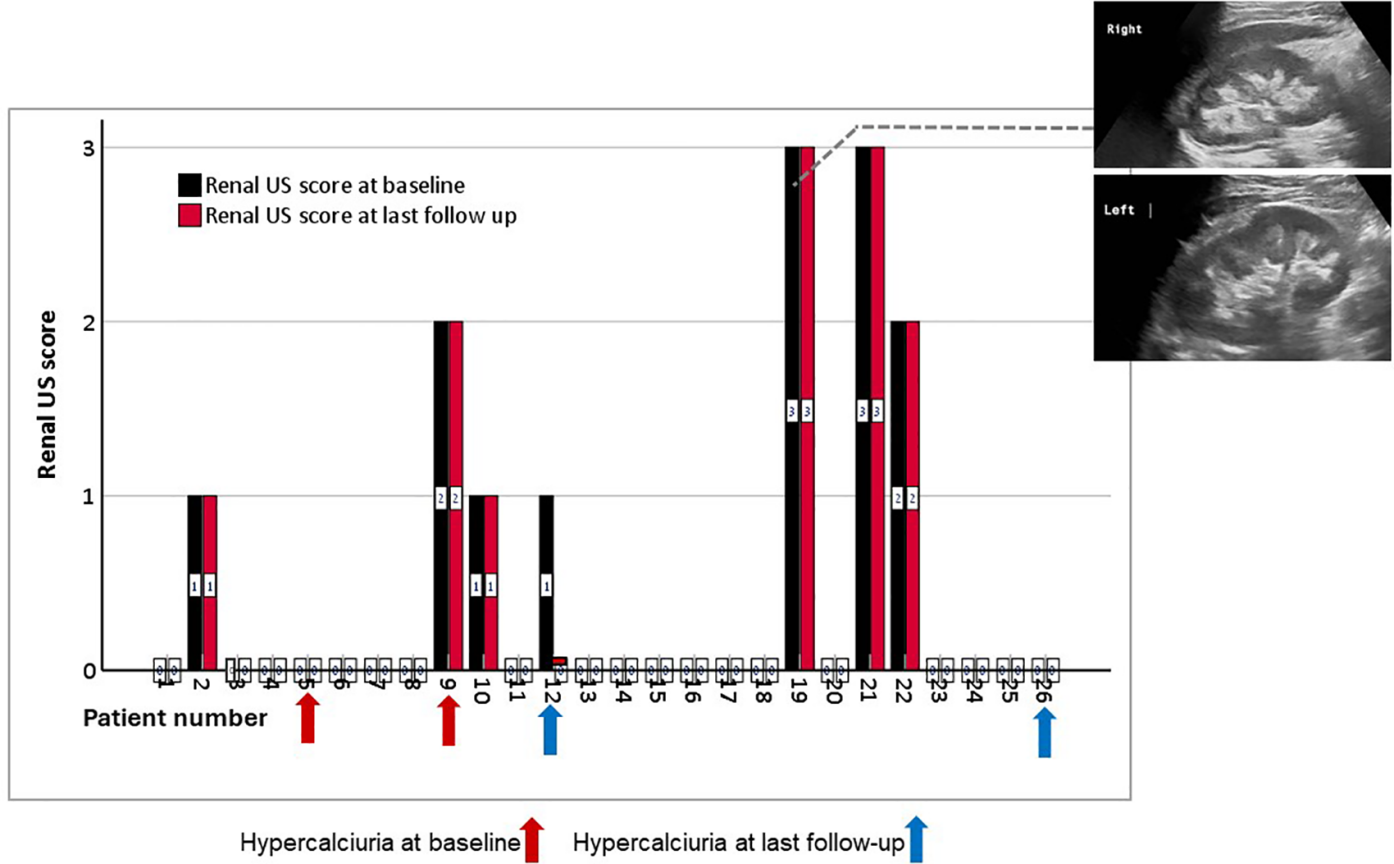
Bar chart of NC scores for each of the 26 patients, measured before (black column) and at the last follow up (red column) after burosumab treatment. The arrows represent the patients with hypercalciuria before (red arrows) and after (blue arrows) burosumab initiation. The figure inserts show examples of grade 3 medullary NC in the right and left kidneys of one patient. US, ultrasound.

All 26 patients had kidney ultrasound images at baseline and during follow up (Figure 4). NC was found in 7 (27%) at baseline: grade 1 in 3, grade 2 in 2 and grade 3 in 2, with a total mean ± SD NC score of 1.8 ± 0.34. NC score did not worsen in any of these patients, and no patient developed new-onset NC during the study period. NC disappeared during follow-up in one patient with baseline NC. Neither patient with new-onset HC showed evidence of NC at the end of the follow-up period (Figure 4, blue arrow).

All children had preserved kidney function during follow up, except for one child with a pre-existing dysplastic kidney. The eGFR was stable during the study period: 149.8 ± 38.4 mL/min/1.73 m^2^ at baseline; and 149.4 ± 42.2, 150.3 ± 36.8, and 133.1 ± 18.3 mL/min/1.73 m^2^ at 12, 24, and 36 months, respectively (*p* = NS). No patient was diagnosed with hypertension.

## Discussion

This study of children with XLH treated with burosumab focused on kidney safety parameters, including HC, NC, and kidney function, along with biochemical parameters and rickets. Our findings align with previous studies, confirming burosumab's effectiveness in improving biochemical and bone parameters compared to conventional therapy (10–12). Brener et al. have previously described the positive impact of burosumab on growth, body composition and dental health of children with XLH, some of whom were included in this cohort (23, 24).

Although burosumab is a breakthrough treatment for children with XLH, with a good safety profile, concerns still exist related to its potential effects on vitamin D activation, which may lead on the long-term to adverse events, mainly associated with pathological tissue calcification (25).

Previous conventional therapy for XLH, which included phosphorus supplements and active vitamin D, is associated with severe adverse effects, including secondary hyperparathyroidism. Active vitamin D analogues can prevent hyperparathyroidism but increase the risk of NC, due to their enhancing effects on intestinal calcium reabsorption (26). As mentioned above, FGF23 inhibits CYP27B1, which decreases vitamin D 1-α-hydroxylase activity, and activates CYP24A1, the 24-hydroxylase enzyme. This lowers levels of the active vitamin D metabolite, [1,25(OH)2D] (27). Inhibition of FGF23 may theoretically cause an uncontrolled elevation in 1,25(OH)2D levels, increasing intestinal calcium absorption, leading to hypercalcemia, HC and NC. Also, as FGF23 lowers PTH gene expression and secretion (28), inhibiting FGF23 may cause hyperparathyroidism, which can also lead to hypercalcemia and HC.

In a study by Imel et al. (29) no significant changes in urinary calcium excretion or progression in NC were observed in adults with XLH treated with burosumab for 1 year. However, two of these patients had an increase in coronary artery or aortic valve calcification. In a phase 3 trial, 61 patients (aged 1–12) received either burosumab or conventional therapy, for 64 weeks (12). NC did not worsen, and no new NC cases occurred. However, in a multicenter clinical trial on the efficacy and safety of burosumab treatment (10, 30), 52 pediatric patients with XLH, (aged 5–12), were followed for 64 weeks with extension period to a total of 160 weeks. There were not significant changes in serum calcium, urinary calcium, and serum PTH. After 64 weeks of follow-up 2 patients had a decrease, and 6 patients had an increase in NC score. At the end of 160 weeks of follow-up, NC scores remained stable in 39 children, decrease by −1 in 3, increase by +1 in 9 and by +2 in 1. Therefore, the concerns related to NC dynamics under burosumab treatment remains relevant.

Published guidelines include the recommendation to follow up kidney related parameters in patients with XLH treated with burosumab including HC and NC (2).

The “real world” effectiveness of burosumab treatment was first published by Paloian et al. (31). In that single-center cohort of 12 patients, followed for 1 year, burosumab treatment was superior to conventional therapy. High average PTH levels improved significantly after switching to burosumab. Four patients had hyperparathyroidism while treated with convention treatment, which improved in all under burosumab treatment. The average urinary calcium excretion was normal at baseline and remained within normal limits during follow up. NC was diagnosed at baseline in 2/12 patients. No significant changes occurred during treatment and none of the patients were recorded as having new onset NC. Recently, Paloian et al. (32) published a series of 13 patients treated with burosumab after a mean of 4.2 ± 4 years of conventional therapy. One patient in their cohort developed new-onset NC after 5 years of burosumab therapy.

In our study, 26 patients were followed for a mean 28.4 ± 9.4 months. During the study period, hyperparathyroidism improved and 1,25(OH)2D levels did not increase significantly. The urinary calcium excretion was regularly tested, and mean values remained stable during follow-up. Two patients had HC while treated with conventional therapy, which improved and resolved during burosumab treatment. Two other patients had new onset HC, which was more prominent in only one of them (number 26). We could not find any explanation for her having overt HC, as it was not related to hyperparathyroidism or high 1,25(OH)2D levels and did not improve after burosumab dose reduction. Interestingly, both patients were among the younger patients in the cohort: aged 2.5 and 2.8 years at baseline. To mention, in these two cases, the treating physicians ensured that the samples were collected in fasting condition. One patient had received short-term XLH conventional therapy, and one did not receive any previous therapy. While HC was borderline for age (33) in patient number 12, it was overtly abnormal in patient number 26 [mean U-Ca/Cr values for this patient: 0.5 (range 0.27–0.78) mg/mg; *n* = 8 tests]. This patient underwent a repeat dietary evaluation, during which a reduction in dietary salt and proper hydration were recommended. Additionally, in this patient, we followed urinary citrate levels which were within normal- high levels. Frequent ultrasound follow-up showed no kidney deposits nor kidney stones.

The most important kidney outcome in XLH is the development of NC and its severity. Intratubular calcium deposition can potentially cause tubular obstruction, impaired tubular function, tubular atrophy, and interstitial inflammation/fibrosis, and thus kidney damage (34). A strength of our study is that an experienced single radiologist, who was blinded to the patients’ data, revised all the sonographic imaging using an accepted grading score (22). In contrast to previously published data (10, 30), no patients showed deterioration or developed new onset NC during the mean sonographic follow-up period of 26.0 ± 10.3 months. The two patients with new onset HC did not develop NC at the last follow up. In our cohort, we assume that the low rates of HC, in addition to the lack of deterioration in NC severity, are related to the close surveillance of kidney-related parameters, including PTH, 1,25(OH)2D levels, and urinary calcium excretion. It may also be related to patient education regarding proper hydration, dietary recommendation of low salt diet and lemon consumption (as a source of urinary citrate) to protect against NC and stone formation (35). Moreover, we presume that the positive results are related to the adherence to the guidance of keeping phosphate levels in low-normal range (2). A similar therapeutic approach was reported in a study that showed that complete normalization of serum phosphate is not mandatory for substantial improvement in rickets in pediatric patients with XLH (36).

Our study has a number of limitations. First, its retrospective nature resulting in missing data, such as 1,25(OH)2D levels in some patients and data regarding urinary citrate and sodium excretion. In addition, we have limited data on 24-h urine calcium excretion, as most treating physicians followed the guideline recommend of monitoring urinary Ca/Cr ratio in spot samples (2). However, 2 of 4 patients with HC did have assessment of 24 h urine collections for calcium. It is important to mention that previous studies have demonstrated relatively strong correlations between Ca/Cr ratio in a single spot urine sample and 24-h urinary calcium excretion, particularly in first-morning and evening samples (18). In addition, to overcome this limitation, all patients provided multiple urine samples (mean 7.5 ± 2.4), and to define hypercalciuria, at least two pathological urine spot tests had to be identified. Even though we followed serum creatinine levels routinely (which remained stable), other markers for kidney injury, such as proteinuria and albuminuria were not available for most of the patients. Finally, although the group was relatively large for the reported prevalence of XLH, and similar to the size of previously published clinical trials (10, 12), HC was an infrequent finding. Thus, we could not assess for risk factors associated with HC. Those parameters still need to be assessed in larger cohorts, using a multi-national approach.

In summary, in addition to the previously demonstrated efficacy of burosumab for normalization of serum phosphate and healing of rickets in pediatric XLH, this study shows no evidence for adverse kidney outcomes, including a low incidence of HC and no increase in NC. Maintaining phosphate levels in the lower range of normal, as recommended in the guidelines, as well as close surveillance of kidney safety parameters is important for the prevention of this low, but existing, risk of HC.

## Data Availability

Publicly available datasets were analyzed in this study. This data can be found here: Not applicable.
